# Assessment of depression in people with diabetes attending outpatient clinics for the treatment of foot ulceration

**DOI:** 10.1186/1757-1146-6-S1-O28

**Published:** 2013-05-31

**Authors:** Toni Nash, Vanessa Ireland, Sue Pearson

**Affiliations:** 1 Southern Tasmanian Area Health Service, Hobart, Tasmania, Australia; 2 University of Tasmania & Menzies Centre, Hobart, Tasmania, Australia

## Background

People with diabetes and foot ulceration experience more depressed mood particularly when healing does not occur after prolonged treatment. Those who have depression and diabetes have poorer adherence to self-care or treatment regimes, poor glycaemic control along with a greater risk of diabetes related complications and mortality. Screening for depression has been shown to be effective to determining the severity of depression as well as the type of treatment required.

## Methods

The study required participants to complete a validated self-reporting 9 item Patient Health Questionnaire (PHQ) that provides a diagnosis of major depressive syndrome and continuous severity score. Participants were classified in the depressed category if they had PHQ score ≥5. Group differences were examined using chi-square for categorical variables and *t*-tests for continuous variables.

## Results

A higher proportion of participants had diabetes for >10 years, however no other demographic variables were associated with depression. Of the 60 participants, 31 (51.7%) were categorised as depressed, with 10 (17%) having had a prior diagnosis of depression and 21 (35%) participants had unrecognised cases of depression.

## Conclusion

This study demonstrated the prevalence of depression in people with diabetes and the often hidden impact it has in managing diabetes foot ulceration.

